# Correction to ‘*ALOX5, LPA, MMP9* and *TPO* gene polymorphisms increase atherothrombosis susceptibility in middle-aged Mexicans’

**DOI:** 10.1098/rsos.200167

**Published:** 2020-02-19

**Authors:** Rafael Camacho-Mejorado, Rocío Gómez, Luisa E. Torres-Sánchez, Esther Alhelí Hernández-Tobías, Gino Noris, Carla Santana, Jonathan J. Magaña, Lorena Orozco, Aurora de la Peña-Díaz, María de la Luz Arenas-Sordo, Marco Antonio Meraz-Ríos, Abraham Majluf-Cruz

*R. Soc. open sci.* **7**, 190775. (Published 15 January 2020). (doi:10.1098/rsos.190775)

This correction refers to an error in the affiliation of author ‘María de la Luz Arenas-Sordo’. It should be:

^5^Departmento de Genética, INR, Mexico City, Mexico

This has now been corrected.

