# Supplementary material for: ALOX5, LPA, MMP9 and TPO gene polymorphisms increase atherothrombosis susceptibility in middle-aged Mexicans
Source: R Soc Open Sci. 2020 Jan 15;7(1):190775. doi: 10.1098/rsos.190775 (PMC7029922; doi:10.1098/rsos.190775)
Supplement: Supplementary Table Titles [file rsos190775supp2.docx]

**Table captions.**

**Table S1.**

Title. Classical risk factors criteria and its indicators and parameters.

Footnote. mg/dL: milligrams/decilitre.

**Table S2.**

Title. Information about the Native American populations used as ancestral control.

**Table S3.**

Title. Information on the nine gene polymorphisms explored.

Footnote. ONIM: Online Mendelian Inheritance in Man, UniSTS: Database of sequence-tagged sites (STSs).

**Table S4.**

Title. PCR amplification conditions for the nine gene polymorphisms studied.

**Table S5.**

Title. Allele frequencies of the nine gene polymorphisms studied in the group with clinical manifestations of atherothrombosis.

Footnote. *k*: Number of different alleles; *Fis*: average deviation from Hardy-Weinberg expectation; HWE: Hardy-Weinberg expectation. Bold numbers depict significant differences.

**Table S6.**

Title. Allele frequencies of the nine gene polymorphisms studied in the healthy group.

Footnote. *k*: Number of different alleles; *Fis*: average deviation from Hardy-Weinberg expectation; HWE: Hardy-Weinberg expectation.

**Table S7.**

Title. Genotypic frequencies of the *ALOX5* polymorphism in the study groups.

Footnote. CMA: Clinical manifestations of atherothrombosis; GC: Genomic control. Bold numbers depict the most frequent genotypes.

**Table S8.**

Title. Genotypic frequencies of the *eNOS* polymorphism in the study groups.

Footnote. CMA: Clinical manifestations of atherothrombosis; GC: Genomic control. Bold numbers depict the most frequent genotypes.

**Table S9.**

Title. Genotypic frequencies of the *HMOX* polymorphism in the study groups.

Footnote. CMA: Clinical manifestations of atherothrombosis; GC: Genomic control. Bold numbers depict the most frequent genotypes.

**Table S10.**

Title. Genotypic frequencies of the *IL6* polymorphism in the study groups.

Footnote. CMA: Clinical manifestations of atherothrombosis; GC: Genomic control. Bold numbers depict the most frequent genotypes.

**Table S11.**

Title. Genotypic frequencies of the *LPA* polymorphism in the study groups.

Footnote. CMA: Clinical manifestations of atherothrombosis; GC: Genomic control. Bold numbers depict the most frequent genotypes.

**Table S12.**

Title. Genotypic frequencies of the *MMP9* polymorphism in the study groups.

Footnote. CMA: Clinical manifestations of atherothrombosis; GC: Genomic control. Bold numbers depict the most frequent genotypes.

**Table S13.**

Title. Genotypic frequencies of the *NOX4* polymorphism in the study groups.

Footnote. CMA: Clinical manifestations of atherothrombosis; GC: Genomic control. Bold numbers depict the most frequent genotypes.

**Table S14.**

Title. Genotypic frequencies of the *TPO* polymorphism in the study groups.

Footnote. CMA: Clinical manifestations of atherothrombosis; GC: Genomic control. Bold numbers depict the most frequent genotypes.

**Table S15.**

Title. Genotypic frequencies of the *vWA* polymorphism in the study groups.

Footnote. CMA: Clinical manifestations of atherothrombosis; GC: Genomic control. Bold numbers depict the most frequent genotypes.

**Table S16.**

Title. Allele frequencies of the nine gene polymorphisms studied in the genomic control.

Footnote. *k*: Number of different alleles; *Fis*: average deviation from Hardy-Weinberg expectation; HWE: Hardy-Weinberg expectation.

**Table S17.**

Title. Allele frequencies of the nine gene polymorphisms studied in the ancestral control (Mayas).

Footnote. *k*: Number of different alleles; *Fis*: average deviation from Hardy-Weinberg expectation; HWE: Hardy-Weinberg expectation. Bold numbers depict significant differences.

**Table S18.**

Title. Allele frequencies of the nine gene polymorphisms studied in the ancestral control (Mazahuas).

Footnote. *k*: Number of different alleles; *Fis*: average deviation from Hardy-Weinberg expectation; HWE: Hardy-Weinberg expectation. Bold numbers depict significant differences.

**Table S19.**

Title. Allele frequencies of the nine gene polymorphisms studied in the ancestral control (Me’Phaas).

Footnote. *k*: Number of different alleles; *Fis*: average deviation from Hardy-Weinberg expectation; HWE: Hardy-Weinberg expectation. Bold numbers depict significant differences.

**Table S20.**

Title. Allele frequencies of the nine gene polymorphisms studied in the ancestral control (Mixes).

Footnote. *k*: Number of different alleles; *Fis*: average deviation from Hardy-Weinberg expectation; HWE: Hardy-Weinberg expectation. Bold numbers depict significant differences.

**Table S21.**

Title. Allele frequencies of the nine gene polymorphisms studied in the ancestral control (Nahuas).

Footnote. *k*: Number of different alleles; *Fis*: average deviation from Hardy-Weinberg expectation; HWE: Hardy-Weinberg expectation. Bold numbers depict significant differences.

**Table S22.**

Title. Allele frequencies of the nine gene polymorphisms studied in the ancestral control (Rarámuris).

Footnote. *k*: Number of different alleles; *Fis*: average deviation from Hardy-Weinberg expectation; HWE: Hardy-Weinberg expectation. Bold numbers depict significant differences.

**Table S23.**

Title. Allele frequencies of the nine gene polymorphisms studied in the ancestral control (Yoremes).

Footnote. *k*: Number of different alleles; *Fis*: average deviation from Hardy-Weinberg expectation; HWE: Hardy-Weinberg expectation.

References

1. Stangl K, Cascorbi I, Laule M, Klein T, Stangl V, Rost S, *et al*. 2000. High CA repeat numbers in intron 13 of the endothelial nitric oxide synthase gene and increased risk of coronary artery disease. *Pharmacogenetics* **10**, 133-140.

2. Lorenz M, Hewing B, Hui J, Zepp A, Baumann G, Bindereif A, *et al*. 2007.Alternative splicing in intron 13 of the human eNOS gene: a potential mechanism for regulating eNOS activity. *FASEB J.* **21*,*** 1556-1564. (doi: 10.1096/fj.06-7434com)

3. Rosby O, Berg K. 2000. LPA gene: interaction between the apolipoprotein(a) size ('kringle IV' repeat) polymorphism and a pentanucleotide repeat polymorphism influences Lp(a) lipoprotein level. *J Intern Med.* **247**,139-152.

4. Meraz-Rios MA, Majluf-Cruz A, Santana C, Noris G, Camacho-Mejorado R, Acosta-Saavedra LC, *et al*. 2014 Association of vWA and TPOX polymorphisms with venous thrombosis in Mexican mestizos. *Biomed Res Int.* 697689.(doi: 10.1155/2014/697689)

5. Qiao H, Sai X, Gai L, Huang G, Chen X, Tu X, *et al.* 2014. Association Between Heme Oxygenase 1 Gene Promoter Polymorphisms and Susceptibility to Coronary Artery Disease: A HuGE Review and Meta-Analysis. *Am J Epidemiol.* **179**, 1039-1048. (doi: 10.1093/aje/kwu024)

6. Schillinger M, Exner M, Mlekusch W, Domanovits H, Huber K, Mannhalter C, *et al.* 2002. Heme oxygenase-1 gene promoter polymorphism is associated with abdominal aortic aneurysm. *Thromb Res.* **2002***,* 131-136. (doi: 10.1016/s0049-3848(02)00100-7)

7. Magana JJ, Gomez R, Cisneros B, Casas L, Valdes-Flores M. 2008. Association of interleukin-6 gene polymorphisms with bone mineral density in Mexican women. *Arch Med Res.* **39**, 618-624. (doi: 10.1016/j.arcmed.2008.05.006)

8. Tsukamoto K, Haruta K, Shiba T, Emi M. 1998. Isolation and mapping of a polymorphic CA repeat sequence at the human interleukin 6 locus. *J Hum Genet.* **43**, 71-72. (doi: 10.1007/s100380050042)

9. Uhlemann AC, Szlezak NA, Vonthein R, Tomiuk J, Emmer SA, Lell B, *et al.* 2004. DNA phasing by TA dinucleotide microsatellite length determines in vitro and in vivo expression of the gp91phox subunit of NADPH oxidase and mediates protection against severe malaria. *J Infect Dis.* **189**, 2227-2234.(doi: 10.1086/421242)

10. Maeda S, Haneda M, Guo B, Koya D, Hayashi K, Sugimoto T, *et al.* 2001. Dinucleotide repeat polymorphisms of matrix metalloproteinase-9 gene is associated with diabetic nephropathy. *Kidney Int.* **60**, 1428-1434.(doi: 10.1046/j.1523-1755.2001.00945.x)

11. Dwyer JH, Allayee H, Dwyer KM, Fan J, Wu H, Mar R, *et al*. 2004. Arachidona-te 5-lipoxygenase promoter genotype, dietary arachidonic acid, and atherosclerosis. *N Engl J Med.* **350**, 29-37. (doi: 10.1056/NEJMoa025079)

12. Schentrup AM, Allayee H, Lima JJ, Johnson JA, Langaee TY.2009. Genotyping the GGGCGG tandem repeat promoter polymorphism in the 5-lipoxygenase enzyme gene (ALOX5) by pyrosequencing assay. *Genet Test Mol Biomarkers.* **13**, 361-365. (doi: 10.1089/gtmb.2008.0103)
